# Outcomes comparison of robotic-assisted versus laparoscopic and open surgery for patients undergoing rectal cancer resection with concurrent stoma creation

**DOI:** 10.1007/s00464-024-10996-4

**Published:** 2024-06-28

**Authors:** Robert N. Goldstone, Todd Francone, Gediwon Milky, I-Fan Shih, Hannah Bossie, Yanli Li, Rocco Ricciardi

**Affiliations:** 1 Department of Gastrointestinal and Surgical Oncology, Massachusetts General Hospital, Harvard Medical School, 15 Parkman Street WACC 460, Boston, MA 02114 USA; 2 Intuitive Surgical, Sunnyvale, CA USA

**Keywords:** Rectal cancer, Robotic surgery, Proctectomy, Ileostomy, Colostomy

## Abstract

**Background:**

Despite widespread adoption of robotic-assisted surgery (RAS) in rectal cancer resection, there remains limited knowledge of its clinical advantage over laparoscopic (Lap) and open (OS) surgery. We aimed to compare clinical outcomes of RAS with Lap and OS for rectal cancer.

**Methods:**

We identified all patients aged ≥ 18 years who had elective rectal cancer resection requiring temporary or permanent stoma formation from 1/2013 to 12/2020 from the PINC AI™ Healthcare Database. We completed multivariable logistic regression analysis accounting for hospital clustering to compare ileostomy formation between surgical approaches. Next, we built inverse probability of treatment-weighted analyses to compare outcomes for ileostomy and permanent colostomy separately. Outcomes included postoperative complications, in-hospital mortality, discharge to home, reoperation, and 30-day readmission.

**Results:**

A total of 12,787 patients (OS: 5599 [43.8%]; Lap: 2872 [22.5%]; RAS: 4316 [33.7%]) underwent elective rectal cancer resection. Compared to OS, patients who had Lap (OR 1.29, *p* < 0.001) or RAS (OR 1.53, *p* < 0.001) were more likely to have an ileostomy rather than permanent colostomy. In those with ileostomy, RAS was associated with fewer ileus (OR 0.71, *p* < 0.001) and less bleeding (OR 0.50, *p* < 0.001) compared to Lap. In addition, RAS was associated with lower anastomotic leak (OR 0.25, *p* < 0.001), less bleeding (OR 0.51, *p* < 0.001), and fewer blood transfusions (OR 0.70, *p* = 0.022) when compared to OS. In those patients who had permanent colostomy formation, RAS was associated with fewer ileus (OR 0.72, *p* < 0.001), less bleeding (OR 0.78, *p* = 0.021), lower 30-day reoperation (OR 0.49, *p* < 0.001), and higher discharge to home (OR 1.26, *p* = 0.013) than Lap, as well as OS.

**Conclusion:**

Rectal cancer patients treated with RAS were more likely to have an ileostomy rather than a permanent colostomy and more enhanced recovery compared to Lap and OS.

**Supplementary Information:**

The online version contains supplementary material available at 10.1007/s00464-024-10996-4.

With the development and advancement of minimally invasive surgical options, patient outcomes have continued to improve over time. New technologies, including robotic surgical systems, continue to contribute to this evolution of outcomes through enhanced efficiency, reproducibility of approach, advanced visualization, and more precise instrumentation. In the case of robotics, the adoption of these advanced technologies may have occurred at a faster rate than the publication of outcomes data. As the robotic technology has continued to evolve to enable greater multi-quadrant access, integrated advanced vessel sealing and stapling technology, and advanced fluorescence imaging, it is important that we have an updated perspective on how these features translate to value for patients as well as providers.

For many types of operations, robotic approaches have become surgeons’ preferred techniques. For example, in the area of colorectal surgery, the adoption of robotic technology has become particularly valuable in pelvic procedures. At this time, there is an established practical role for robotic techniques when operating in a challenging pelvis or in patients with difficult rectal tumors. In fact, in prospective randomized trials, the advantages of robotics have been demonstrated at centers with significant robotics experience which observed less bleeding, fewer complications, reduced surgical trauma, and enhanced postoperative recovery as compared to laparoscopic surgery [1]. At high volume robotics centers, patients are treated by surgeons with considerable expertise in robotics. However, many patients receive their care at facilities with varying robotics expertise. As the adoption and diffusion of robotics technology continue, it is unclear if patients don’t receive better benefits from a robotics platform, as prior studies reported [2], across healthcare settings.

Moreover, outcomes may vary between patients with ileostomy formation and those with permanent colostomy formation [3, 4], possibly confounding the effect of robotic approach when left unaccounted for. In order to demonstrate population-wide outcomes gains from robotic technology, the advantages and effectiveness of robotic techniques would benefit from pragmatic controlled trials [5]. An alternative approach may be gained from real-world observational data to determine the effectiveness of robotics in various health care settings. In this study, we analyzed a real-world data representing 25% of US in-patient admissions to understand the benefits of robotics in rectal cancer surgery for patients with ileostomy formation and patients with colostomy formation. We hypothesized that robotic rectal resections are associated with fewer conversions, shorter length of stay, fewer complication rates, higher rate of discharge to home, and similar in-hospital mortality as compared to laparoscopic and open surgical approaches.

## Methods

### Study design

A retrospective cohort study was conducted using the PINC AI™ Healthcare Database, formerly known as the Premier Healthcare Database. The database contains service-level information for more than 279 million patients accounting for nearly 8 million hospital-based in-patient admissions per year (representing 25% of U.S. in-patient admissions). The database includes de-identified, HIPPA compliant healthcare information about hospital and visit characteristics, physician characteristics, and patient characteristics from standard hospital discharge billing files [6]. The study was exempted from review by an institutional review board in accordance with 45 CFR §46.

The study population included patients aged 18 years or older who underwent elective in-patient rectal cancer resections with an ileostomy or colostomy formation between January 1, 2013, through December 30, 2020. By including only patients with rectal cancer and stoma (either permanent or temporary), we have reduced the heterogeneity of upper rectal cancer which is less challenging to resect and more commonly treated with primary anastomosis. We hypothesize lower rectal tumors are more likely to benefit from robotic procedures. Furthermore, we excluded patients who had repeat rectal resections during the study period, had discharge Disease-Related Group (DRG) code not related to colorectal surgery (329, 330, 331, 332, 333, 334), had procedure codes for both ileostomy and colostomy formations, and had missing/unknown operative time or missing demographic characteristics. The International Classification of Diseases, Ninth or Tenth Revision (ICD-9 or ICD-10) codes, the Current Procedural Terminology (CPT) codes, and hospital billing records shown in eTable 1 were queried for to identify rectal cancer diagnosis, surgical procedures, and corresponding surgical approach for each patient. Patients were categorized into robotic-assisted (RAS), laparoscopic (Lap), or open (OS) group according to their surgical approach.

### Outcome variables

The primary outcome for this study was postoperative complications including anastomotic leak, ileus, bleeding/transfusion, surgical site infection, and urinary retention censored at discharge date. Secondary outcomes included length of hospital stay, in-hospital mortality, discharge to home with or without homecare, index and 30-day reoperation, and 30-day readmission. In addition, conversion to open approach was assessed for RAS versus Lap comparisons. In-hospital mortality and discharge to home status were assessed from the discharge status variable in the patient demographic table. Index reoperation status was defined based on record for surgery time billing 1 day after the index surgery through discharge date. Thirty-day reoperation was defined using the Healthcare Cost and Utilization Project (HCUP) narrow surgery codes [7, 8] along with billing record for surgery time in the 30-day postdischarge period. In-patient hospital admission within the 30 days after discharge was defined by 30-day readmission status. Diagnosis and procedure codes used to identify postoperative complications are shown in eTable 1.

### Study covariates

Patient, surgeon, and hospital characteristics were used as covariates in the analysis. Patient characteristics included age, sex, race/ethnicity, marital status, payor type, Charlson Comorbidity Index (CCI), obesity/overweight status, smoking history, and year of surgery. Surgeon characteristics included surgeon specialty and surgeon procedure volume. Hospital characteristics included teaching status, hospital bed size, geographic region, rural/urban location, and hospital procedure volume. Surgeon volume was calculated as the number of colorectal procedures the surgeon had performed using same surgical approach in the previous one-year period. Surgeon procedure volume was categorized as low volume, medium volume, or high volume based on tertiles distribution. Hospital procedure volumes were calculated as the number of colorectal procedures performed in the previous one-year period. Hospital procedure volume was categorized as low volume, medium volume, or high volume using tertiles as cutoff points.

### Statistical analysis

Sample descriptive statistical analysis on patient, surgeon, and hospital related characteristics was conducted by surgical approach. Multivariable logistic regression analysis accounting for hospital-level clustering was conducted to examine association between surgical approach and ileostomy formation. Outcomes were compared between RAS versus Lap, and open group for the ileostomy and colostomy cohorts separately. To adjust for difference between groups and minimize selection bias, stabilized inverse probability of treatment weighting (IPTW) was performed when comparing outcomes for each cohort. Adjustment with IPTW creates a synthetic sample which is independent of covariates and allows for estimation of unbiased average treatment effects [9]. To ensure no residual differences exist between groups, baseline characteristics were compared before and after IPTW adjustment using Chi-square test for categorical variables and Wilcoxon rank-sum test for continuous variable. Any variable with residual between groups was included as covariate in the outcome regression model. All analyses were performed using R statistical software (version 4.2.2) [10]. For all analyses, *p* < 0.05 was considered statistically significant.

## Results

### Characteristics

**Fig. 1:**
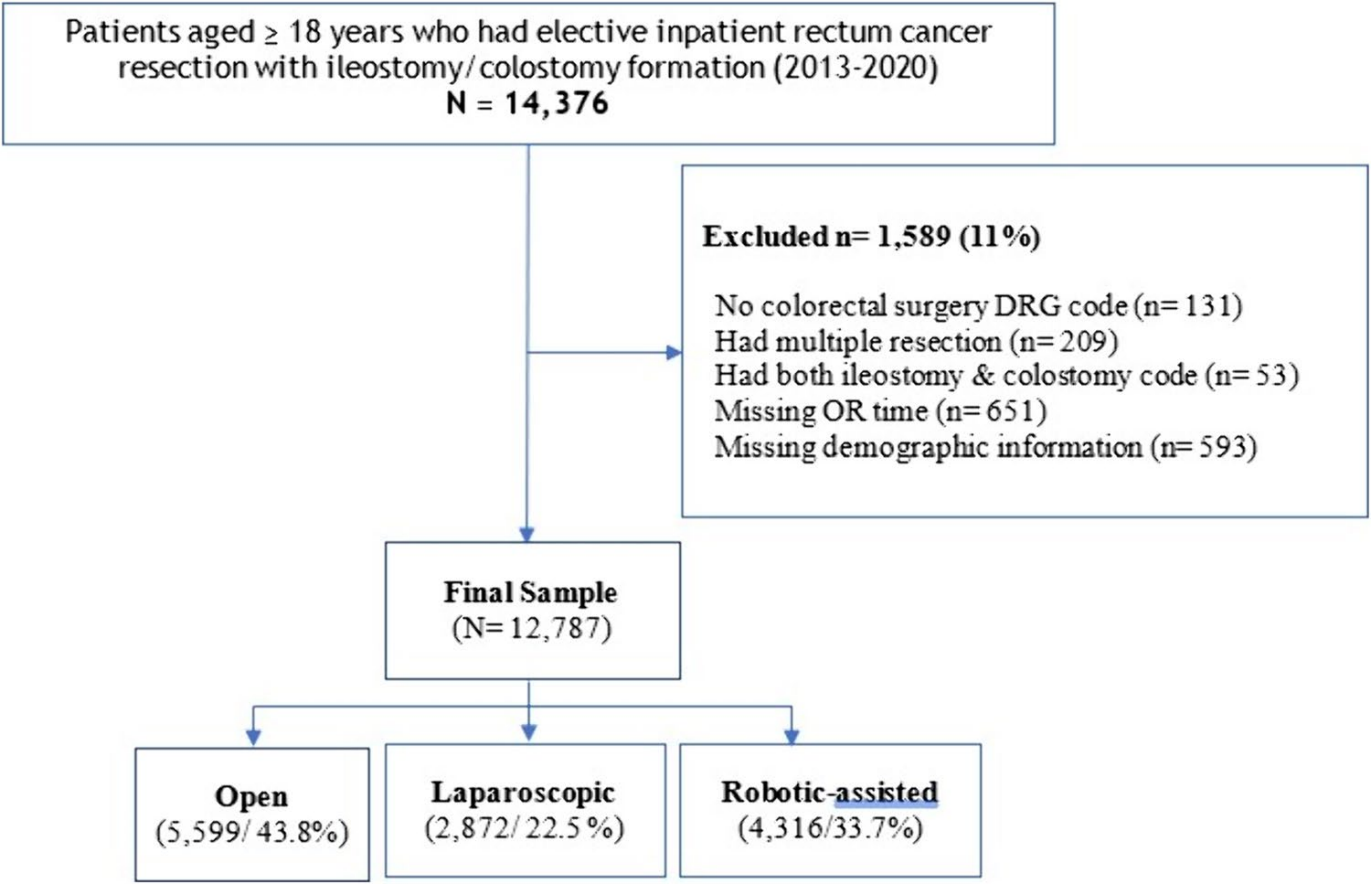
Sample selection flowchart

There were a total of 14,376 patients aged ≥ 18 years old who had elective in-patient rectal cancer resections and had stoma formation in the database. After applying exclusion criteria, the final sample consisted of 12,787 patients, of whom 5599 (43.8%) had open surgery, 2872 (22.5%) had Lap, and 4316 (33.7%) had RAS (Fig. 1). As shown in Table 1, a majority of the sample was male (8087 [63.2%]), and 5839 (45.7%) patients had a Charlson’s comorbidity score of 0, excluding rectal cancer diagnosis. Overall, more than one-half of the patients (7275 [56.9%]) had colostomy formation performed with the resection, except for RAS group in which colostomy formation was performed for 2,111 (48.9%) patients. In a multivariable logistic regression analysis (eTable 2), patients who had Lap (OR 1.29, 95% CI 1.16–1.43, *p* < 0.001), and those who had RAS (OR 1.53, 95% CI 1.39–1.69, *p* < 0.001) were more likely to have an ileostomy rather than colostomy as compared to open surgery.

**Table 1:** Descriptive statistics of patient, surgeon, and hospital characteristics of the study sample

Characteristics	Overall, *N* = 12,787	Open, *N* = 5599	Lap, *N* = 2872	RAS, *N* = 4316	*p*
*Type of stoma*					** < .001**
Colostomy	7275 (56.9)	3549 (63.4)	1615 (56.2)	2111 (48.9)	
Ileostomy	5512 (43.1)	2050 (36.6)	1257 (43.8)	2205 (51.1)	
*Age (years)*					** < .001**
18–44	884 (6.9)	339 (6.1)	202 (7.0)	343 (7.9)	
45–54	2566 (20.1)	1045 (18.7)	592 (20.6)	929 (21.5)	
55–64	3759 (29.4)	1630 (29.1)	826 (28.8)	1303 (30.2)	
65+	5578 (43.6)	2585 (46.2)	1252 (43.6)	1741 (40.3)	
*Sex*					0.230
Female	4700 (36.8)	2086 (37.3)	1072 (37.3)	1542 (35.7)	
Male	8087 (63.2)	3513 (62.7)	1800 (62.7)	2774 (64.3)	
*Marital status*					** < .001**
Single	4969 (38.9)	2277 (40.7)	1064 (37.0)	1628 (37.7)	
Married	6841 (53.5)	2829 (50.5)	1572 (54.7)	2440 (56.5)	
Other	977 (7.6)	493 (8.8)	236 (8.2)	248 (5.7)	
*Race/ethnicity*					**0.009**
Black	1002 (7.8)	457 (8.2)	212 (7.4)	333 (7.7)	
White	10,086 (78.9)	4346 (77.6)	2264 (78.8)	3476 (80.5)	
Hispanic	747 (5.8)	350 (6.3)	182 (6.3)	215 (5.0)	
Other	952 (7.4)	446 (8.0)	214 (7.5)	292 (6.8)	
*Obese*					
Obese	1981 (15.5)	809 (14.4)	457 (15.9)	715 (16.6)	**0.012**
Not obese	10,806 (84.5)	2415 (84.1)	4790 (85.6)	3601 (83.4)	
*Smoking*					
Smoking	5129 (40.1)	2262 (40.4)	1119 (39.0)	1748 (40.5)	0.360
Not smoking	7658 (59.9)	1753 (61.0)	3337 (59.6)	2568 (59.5)	
*CCI score*					** < .001**
0	5839 (45.7)	2413 (43.1)	1368 (47.6)	2058 (47.7)	
1–2	3616 (28.3)	1576 (28.1)	776 (27.0)	1264 (29.3)	
3–4	801 (6.3)	370 (6.6)	172 (6.0)	259 (6.0)	
5 +	2531 (19.8)	1240 (22.1)	556 (19.4)	735 (17.0)	
*Payor type*					** < .001**
Commercial	5253 (41.1)	2082 (37.2)	1199 (41.7)	1972 (45.7)	
Medicare	5581 (43.6)	2603 (46.5)	1255 (43.7)	1723 (39.9)	
Medicaid	1266 (9.9)	602 (10.8)	262 (9.1)	402 (9.3)	
Other	687 (5.4)	312 (5.6)	156 (5.4)	219 (5.1)	
*Surgeon volume*					** < .001**
Low^a^	3996 (31.3)	1649 (29.5)	916 (31.9)	1431 (33.2)	
Medium^b^	4251 (33.2)	1825 (32.6)	958 (33.4)	1468 (34.0)	
High^c^	4540 (35.5)	2125 (38.0)	998 (34.7)	1417 (32.8)	
*Surgeon specialty*					** < .001**
Colorectal	6281 (49.1)	2394 (42.8)	1371 (47.7)	2516 (58.3)	
General	4818 (37.7)	2453 (43.8)	1096 (38.2)	1269 (29.4)	
Other	1688 (13.2)	752 (13.4)	405 (14.1)	531 (12.3)	
*Hospital volume*^d^					** < .001**
Low	3690 (28.9)	1996 (35.6)	860 (29.9)	834 (19.3)	
Medium	4270 (33.4)	1791 (32.0)	941 (32.8)	1538 (35.6)	
High	4827 (37.7)	1812 (32.4)	1071 (37.3)	1944 (45.0)	
*Teaching hospital*					
Yes	7430 (58.1)	3155 (56.3)	1586 (55.2)	2689 (62.3)	** < .001**
No	5357 (41.9)	1286 (44.8)	2444 (43.7)	1627 (37.7)	
*Hospital bed size*					**0.002**
000–300	2769 (21.7)	1267 (22.6)	642 (22.4)	860 (19.9)	
300–499	4130 (32.3)	1844 (32.9)	897 (31.2)	1389 (32.2)	
500 +	5888 (46.0)	2488 (44.4)	1333 (46.4)	2067 (47.9)	
*Urban hospital*					** < .001**
Urban	11,806 (92.3)	5055 (90.3)	2650 (92.3)	4101 (95.0)	
Rural	981 (7.7)	544 (9.7)	222 (7.7)	215 (5.0)	
*Hospital region*					** < .001**
Midwest	3012 (23.6)	1268 (22.6)	629 (21.9)	1115 (25.8)	
Northeast	1748 (13.7)	616 (11.0)	447 (15.6)	685 (15.9)	
South	5903 (46.2)	2718 (48.5)	1204 (41.9)	1981 (45.9)	
West	2124 (16.6)	997 (17.8)	592 (20.6)	535 (12.4)	
*Surgery year*					** < .001**
2013	1969 (15.4)	1195 (21.3)	447 (15.6)	327 (7.6)	
2014	2161 (16.9)	1111 (19.8)	558 (19.4)	492 (11.4)	
2015	1979 (15.5)	941 (16.8)	508 (17.7)	530 (12.3)	
2016	1423 (11.1)	652 (11.6)	305 (10.6)	466 (10.8)	
2017	1439 (11.3)	551 (9.8)	331 (11.5)	557 (12.9)	
2018	1388 (10.9)	433 (7.7)	291 (10.1)	664 (15.4)	
2019	1331 (10.4)	424 (7.6)	253 (8.8)	654 (15.2)	
2020	1097 (8.6)	292 (5.2)	179 (6.2)	626 (14.5)	

*RAS* robotic-assisted surgery, *Lap* laparoscopic surgery, *CCI* Charlson’s comorbidity index

^a^Low refers to ≤ 9 robotic cases, ≤ 5 laparoscopic cases, or ≤ 14 open cases for the respective surgical approaches

^b^Medium refers to 10 to 29 robotic cases, 6 to 22 laparoscopic cases, or 15 to 37 open cases for the respective surgical approaches

^c^High refers to ≥ 30 robotic cases, ≥ 23 laparoscopic cases, and ≥ 38 open cases for the respective surgical approaches

^d^Low refers to ≤ 162 cases, medium refers to 163 to 323 cases, and high refers to ≥ 324 cases

### Ileostomy as component of resection

Baseline characteristics of rectal resection patients with ileostomy formation by surgical approach before and after IPTW adjustment are presented in eTable 3 (RAS vs Lap) and eTable 4 (RAS vs Open). There was residual difference in surgeon volume and hospital volume between RAS and Lap after IPTW, and hence, these variables were included in the outcome regression analyses comparing RAS versus Lap. All baseline characteristics were balanced between RAS and Open after IPTW. Results for the IPTW adjusted analyses of postoperative outcomes are presented in Table 2. Length of hospital stay was approximately 1-day shorter among patients who had RAS as compared to Lap (adjusted mean difference [AMD] = − 0.80 days, 95% CI − 1.08 to − 0.53, *p* < 0.001) and OS (AMD = − 1.11 days, 95% CI − 1.35 to − 0.87, *p* < 0.001). As compared to patients who had Lap, those who had RAS were 70% less likely to have open conversion (OR 0.30, 95% CI 0.24–0.37, *p* < 0.001), and had less likelihood of postoperative ileus (OR 0.71, 95% CI 0.60–0.85, *p* < 0.001) and bleeding (OR 0.50, 95% CI 0.39–0.64, *p* < 0.001). As compared to patients who had OS, those who had RAS had less likelihood of anastomotic leak (OR 0.25, 95% CI 0.16–0.38, *p* < 0.001), less likelihood of bleeding (OR 0.51, 95% CI 0.40–0.65, *p* < 0.001), and less likelihood of blood transfusion (OR 0.70, 95% CI 0.51–0.95, *p* = 0.022).

**Table 2:** Inverse probability of weighting adjusted postoperative clinical outcomes of robotic-assisted versus laparoscopic and open surgery among patients who had rectum resection with ileostomy formation

Outcome	RAS versus lap^a^				RAS versus open			
	Lap, N = 1835	RAS, N = 1627	O.R. (95% CI) *RAS vs Lap*	*p* value	Open, N = 2459	RAS, N = 1796	O.R. (95% CI) *RAS vs. Open*	*p* value
Conversion, *n* (%)	412 (22.5)	127 (7.8)	0.30 (0.24, 0.37)	** < .001**	–	–	–	–
LOS, mean (95% CI)	6.88 (6.67–7.10)^b^	6.08 (5.88–6.28)^b^	−0.80 (−1.08, −0.53)^b^	** < .001**	7.24 (7.07–7.41)^b^	6.13 (5.96–6.30)^b^	−1.11 (−1.35, −0.87)^b^	** < .001**
Anastomotic leak, *n* (%)	32 (1.8)	24 (1.5)	0.90 (0.52, 1.53)	0.690	125 (5.1)	24 (1.3)	0.25 (0.16, 0.38)	** < .001**
Ileus, *n* (%)	384 (20.9)	253 (15.6)	0.71 (0.60, 0.85)	** < .001**	401 (16.3)	271 (15.1)	0.91 (0.77, 1.08)	0.300
Bleeding, *n* (%)	207 (11.3)	95 (5.8)	0.50 (0.39, 0.64)	** < .001**	252 (10.2)	100 (5.5)	0.51 (0.40, 0.65)	** < .001**
Transfusion, *n* (%)	57 (3.1)	61 (3.8)	1.18 (0.82, 1.71)	0.370	127 (5.1)	66 (3.7)	0.70 (0.51, 0.95)	**0.022**
SSI, *n* (%)	74 (4.0)	43 (2.6)	0.69 (0.46, 1.00)	0.054	101 (4.1)	54 (3.0)	0.72 (0.51, 1.00)	0.056
Urinary retention, *n* (%)	128 (7.0)	108 (6.7)	0.96 (0.73, 1.25)	0.770	162 (6.6)	110 (6.1)	0.93 (0.72, 1.19)	0.550
Index reoperation, *n* (%)	73 (4.0)	65 (4.0)	0.98 (0.69, 1.38)	0.900	100 (4.1)	77 (4.3)	1.05 (0.78, 1.43)	0.730
30-day reoperation, *n* (%)	32 (1.7)	26 (1.6)	0.92 (0.54, 1.55)	0.750	33 (1.3)	30 (1.7)	1.28 (0.78, 2.11)	0.330
In-hospital Mortality, *n* (%)	8 (0.4)	9 (0.5)	1.15 (0.43, 3.12)	0.780	11 (0.5)	16 (0.9)	1.94 (0.91, 4.27)	0.087
Discharge, home, *n* (%)	1710 (93.1)	1537 (94.5)	1.28 (0.96, 1.70)	0.091	2300 (93.5)	1690 (94.1)	1.12 (0.87, 1.44)	0.400
30-day readmission, *n* (%)	348 (18.9)	284 (17.5)	0.93 (0.78, 1.11)	0.410	470 (19.1)	335 (18.7)	0.97 (0.83, 1.13)	0.700

*Lap* laparoscopic surgery, *RAS* robotic-assisted surgery, *LOS* length of stay, *SSI* surgical site infection

All regression analyses were weighted by inverse probability of treatment weights (IPTW) estimated using random effects logistic regression that accounts for hospital clustering including the variables: age, sex, marital status, race/ethnicity, obesity, smoking status, Charlson comorbidity, payor type, hospital teaching status, bed size, region, urban/rural location, hospital volume, surgeon specialty, surgeon volume, and surgery year

^a^Surgeon volume and hospital volume were not balanced after IPTW; hence, it is included as covariate in outcome regression models

^b^Mean difference (95% CI)

### Colostomy as component of resection

Baseline characteristics of rectal resection patients with colostomy formation by surgical approach before and after IPTW adjustment are presented in eTable 5 (RAS vs Lap) and eTable 6 (RAS vs Open). All baseline characteristics were balanced between comparison groups after IPTW. Results for the IPTW adjusted analyses of postoperative outcomes among colostomy cohort are presented in Table 3. Length of hospital stay for patients who had RAS was approximately 0.75 days shorter than Lap patients (AMD = − 0.74 days, 95% CI − 1.06 to − 0.43, *p* < 0.001) and approximately 1.5 days shorter than OS patients (AMD = − 1.53 days, 95% CI − 1.79 to − 1.28, *p* < 0.001). As compared to patients who had Lap, those who had RAS were 71% less likely to have open conversion (OR 0.29, 95% CI 0.23–0.35, *p* < 0.001), had less likelihoods of postoperative ileus (OR 0.72, 95% CI 0.61–0.86, p < 0.001), bleeding (OR 0.78, 95% CI 0.63–0.96, *p* = 0.021), and 30-day reoperation (OR 0.49, 95% CI 0.34–0.70, *p* < 0.001), and higher likelihood of being discharged to home (OR 1.26, 95% CI 1.05–1.51, *p* = 0.013). As compared to patients who had OS, those who had RAS had less likelihood of postoperative ileus (OR 0.76, 95% CI 0.66–0.88, *p* < 0.001), bleeding (OR 0.68, 95% CI 0.57–0.80, *p* < 0.001), blood transfusion (OR 0.47, 95% CI 0.39–0.56, *p* < 0.001), and surgical site infection (OR 0.74, 95% CI 0.56–0.98, *p* = 0.036) and higher likelihood of discharge to home (OR 1.46, 95% CI 1.26–1.69, *p* < 0.001).

**Table 3:** Inverse probability of weighting adjusted postoperative clinical outcomes of robotic-assisted versus laparoscopic and open surgery among patients who had rectal resection with colostomy formation

Outcome	RAS versus lap				RAS versus open			
	Lap, *N* = 2017	RAS, *N* = 1709	O.R. (95% CI) RAS vs Lap	*p* value	Open, *N* = 3208	RAS, *N* = 2452	O.R. (95% CI) RAS vs. open	*p* value
Conversion, n (%)	453 (22.4)	131 (7.7)	0.29 (0.23, 0.35)	** < .001**	–	–	–	–
LOS, mean (95% CI)	7.80 (7.58–8.03)^a^	7.06 (6.84–7.28)^a^	−0.74 (−1.06, −0.43)^a^	** < .001**	8.57 (8.39–8.75)^a^	7.03 (6.86–7.21)^a^	−1.53 (−1.79, −1.28)^a^	** < .001**
Ileus, n (%)	382 (19.0)	247 (14.5)	0.72 (0.61, 0.86)	** < .001**	591 (18.4)	359 (14.6)	0.76 (0.66, 0.88)	** < .001**
Bleeding, n (%)	235 (11.6)	159 (9.3)	0.78 (0.63, 0.96)	**0.021**	437 (13.6)	236 (9.6)	0.68 (0.57, 0.80)	** < .001**
Transfusion, n (%)	181 (9.0)	124 (7.3)	0.79 (0.62, 1.01)	0.058	439 (13.7)	169 (6.9)	0.47 (0.39, 0.56)	** < .001**
SSI, n (%)	99 (4.9)	67 (3.9)	0.79 (0.57, 1.08)	0.140	142 (4.4)	82 (3.3)	0.74 (0.56, 0.98)	**0.036**
Urinary retention, n (%)	145 (7.2)	120 (7.0)	0.98 (0.76, 1.26)	0.870	202 (6.3)	174 (7.1)	1.14 (0.92, 1.41)	0.220
Index reoperation, n (%)	110 (5.4)	96 (5.6)	1.04 (0.78, 1.37)	0.800	179 (5.6)	126 (5.2)	0.92 (0.73, 1.16)	0.490
30-day reoperation, n (%)	107 (5.3)	46 (2.7)	0.49 (0.34, 0.70)	** < .001**	133 (4.1)	88 (3.6)	0.86 (0.65, 1.13)	0.290
In-hospital Mortality, n(%)	13 (0.7)	7 (0.4)	0.61 (0.23, 1.49)	0.290	27 (0.9)	12 (0.5)	0.59 (0.29, 1.13)	0.130
Discharge to home, n (%)	1681 (83.3)	1474 (86.2)	1.26 (1.05, 1.51)	**0.013**	2602 (81.1)	2114 (86.2)	1.46 (1.26, 1.69)	** < .001**
30-day readmission, n (%)	359 (17.8)	277 (16.2)	0.90 (0.75, 1.06)	0.210	563 (17.6)	404 (16.5)	0.93 (0.81, 1.07)	0.290

*Lap* laparoscopic surgery, *RAS* robotic-assisted surgery, *LOS* length of stay, *SSI* surgical site infection

All regression analyses were weighted by inverse probability of treatment weights (IPTW) estimated using random effects logistic regression that accounts for hospital clustering and including the variables: age, sex, marital status, race/ethnicity, obesity, smoking status, Charlson comorbidity, payor type, hospital teaching status, bed size, region, urban/rural location, hospital volume, surgeon specialty, surgeon volume, and surgery year

^a^Mean difference (95% CI)

## Discussion

Over the past few decades, there have been significant advances in the treatment of rectal cancer ranging from administration of total neoadjuvant therapy with potential for organ preservation to development of variable operative approaches for proctectomy. These operative approaches include total mesorectal excision, transanal total mesorectal excision, and minimally invasive approaches such as laparoscopic or robotic-assisted surgery. Operative technology continues to evolve not only to enhance oncologic outcomes but also for improved patient experience and recovery. Among these approaches, robotic-assisted surgery is becoming the most rapidly accepted among surgeons, especially for complex pelvic surgery such as proctectomy with total mesorectal excision. This presumption is confirmed in our large retrospective study which demonstrates that RAS was utilized for oncologic proctectomy for only 16.6% of cases in 2013 but rose to 57.1% of cases in 2020 (e-Fig. 1).

One of the most desired outcomes for surgical management of rectal cancer is that of sphincter preservation and avoidance of a permanent colostomy. However, restoration of intestinal continuity may be quite difficult for very low rectal cancers, especially in obese patients or male patients with a narrow pelvis. In this study, we found that patients who underwent RAS had significantly greater rates of primary anastomosis with ileostomy as compared to both open and laparoscopic approaches which had rates greater than 50% for permanent colostomy creation. A common assumption is that robotic surgery helps aid deep pelvic dissection due to improved instrumental dexterity/articulation, stable camera platform, and magnified 3-dimensional imaging with enhanced depth perception.

In this study, we identified significant intraoperative and postoperative advantages for RAS proctectomy compared to both open and laparoscopic proctectomy. The most discussed and studied intraoperative impact of RAS proctectomy for the management of rectal cancer as compared to laparoscopic surgery is need for conversion to open surgery. A large meta-analysis completed by Simillis et al. in 2019 found no significant difference in rate of conversion to open surgery among these approaches [11]. However, it may be difficult to generalize the results of the study given the heterogeneity of the trials included in the analysis. In contrast, meta-analyses of 6 randomized controlled trials and 5 propensity score-matched case-matched (PSM) studies completed in the same year by Phan et al. found significantly lower rates of conversion to open surgery with robotic surgery as compared to laparoscopic surgery, in a pooled analysis (OR 0.38, 95% CI 0.30–0.46) as well as in separate analyses of the RCT studies (OR 0.28, 95% CI 0.00–0.57) and PSM studies (OR 0.39, 95% CI 0.30–0.47) [12]. Similarly, a meta-analysis of 5 RCTs completed by Prete et al. concluded that RAS was associated with lower rates of conversion to open surgery as compared to laparoscopic surgery (RR = 0.58, 95% 0.34–0.97) [13]. Our study shows a significant reduction in the likelihood of conversion to open surgery for RAS proctectomy compared to laparoscopic among patients who received a colostomy or ileostomy. This finding is important as it included larger sample size than the prior mentioned studies and may be more representative of the diverse surgical practice in the USA given the database employed.

Other commonly questioned outcomes in the evaluation of operative approaches are those of postoperative morbidity. In our study, we identified that RAS proctectomy with either ileostomy formation or creation of permanent colostomy had a significantly lower likelihood of postoperative ileus and bleeding when compared to laparoscopic surgery, as well as a significantly lower likelihood of postoperative anastomotic leak, bleeding, and blood transfusion when compared to open approaches. Although intraoperative blood loss estimates can be quite skewed and subjective, these results coincide with those from prior smaller studies [11, 14, 15]. Reduction in risk of blood loss and need for transfusion is particularly important in the treatment of cancer, as perioperative blood transfusion has been associated with negative oncologic outcomes including increased risk of tumor recurrence and reduced survival [16, 17].

Additionally, we identified that RAS proctectomy had a significantly reduced likelihood of postoperative ileus and length of hospital stay when compared to laparoscopic and open approaches. These results correspond to results from the largest meta-analysis completed in 2019 by Simillis et al. While minimally invasive surgery has been consistently associated with faster recovery and decreased hospital length of stay compared to open surgery for numerous different operations, it remains unclear why RAS proctectomy has lower length of stay and reduced risk of ileus as compared to laparoscopic. We may surmise that this may be due to decreased analgesic requirements for RAS as shown by Christoffersen et al. [18] and Donlon et al. [19] as well as decreased rates of conversion to open surgery. Regardless of the reasons for this finding, the data are generalizable to the population as a whole.

Interestingly, our analyses also demonstrated that RAS proctectomy with permanent colostomy was associated with a significantly greater likelihood of discharge to home compared to both laparoscopic and open approaches. This is of great importance not only with regards to patient comfort, but also with respect to healthcare expenditure. Our group and others have previously demonstrated that postacute care services and hospital length of stay both strongly impact cost of care [20–22]. Furthermore, prolonged hospital stay while awaiting discharge to skilled nursing facilities or rehabilitation centers can significantly hinder hospital bed availability, which remains a constant burden on large healthcare facilities in urban settings in the post-COVID pandemic.

This study has several limitations related to retrospective analyses. First, the study uses administrative data which lack many of the important medical record details that might reveal whether each variable was coded properly. For example, obese/overweight status is likely to be underestimated in administrative databases, although we do not expect this to be differently assessed between surgical exposures and hence unlikely to change our findings. Additionally, there are many differences in the patient characteristics between each study arm. However, this was corrected for as best possible with the multivariable logistic regression models and IPTW to adjust for the difference between groups and minimize selection bias. Finally, our database did not include oncologic characteristics including tumor size, stage, or use of neoadjuvant therapy which may impact operative approach utilized and postoperative outcomes.

Despite these study limitations, these real-world data which account for approximately 25% of US in-patient admissions per year are more likely representative of current clinical practice and outcomes than that of the previously conducted clinical trials. Clinical trials are typically performed by surgeons with vast laparoscopic or robotic experience which will not reflect actual population-based results which we have identified here. In addition, we included surgeon volume as a covariate in our analysis to control for surgeon learning effect. Our data reveal that for the general population, robotic-assisted surgery may facilitate sphincter preservation and improved total mesorectal excision with lower risk of conversion to an open operation and improved postoperative outcomes/recovery.

## Conclusions

To our knowledge, this represents the largest analysis investigating the difference in outcomes between robotic-assisted, laparoscopic, and open proctectomy with stoma creation for the management of rectal cancer in the USA. Specifically, we identified that RAS proctectomy was associated with greater rates of sphincter preservation and restoration of intestinal continuity as compared to both laparoscopic and open surgeries. Furthermore, RAS proctectomy with proximal diversion or permanent colostomy had lower likelihood of conversion to open, length of hospital stay, postoperative ileus, and bleeding compared to laparoscopic and open approaches. Additionally, robotic-assisted proctectomy was associated with greater likelihood of discharge home with reduced risk of use of postacute care facilities.

### Supplementary Information

Below is the link to the electronic supplementary material.

Supplemental Figure 1. Surgical approach adoption trend
Supplementary file1 (JPG 46 kb)

Supplementary file2 (DOCX 17 kb)

Supplementary file3 (DOCX 18 kb)

Supplementary file4 (DOCX 30 kb)

Supplementary file5 (DOCX 30 kb)

Supplementary file6 (DOCX 30 kb)

Supplementary file7 (DOCX 30 kb)
