# Supplementary figures and images for: Outcomes comparison of robotic-assisted versus laparoscopic and open surgery for patients undergoing rectal cancer resection with concurrent stoma creation
Source: Surg Endosc. 2024 Jun 28;38(8):4550–8. doi: 10.1007/s00464-024-10996-4 (PMC11289169; doi:10.1007/s00464-024-10996-4)

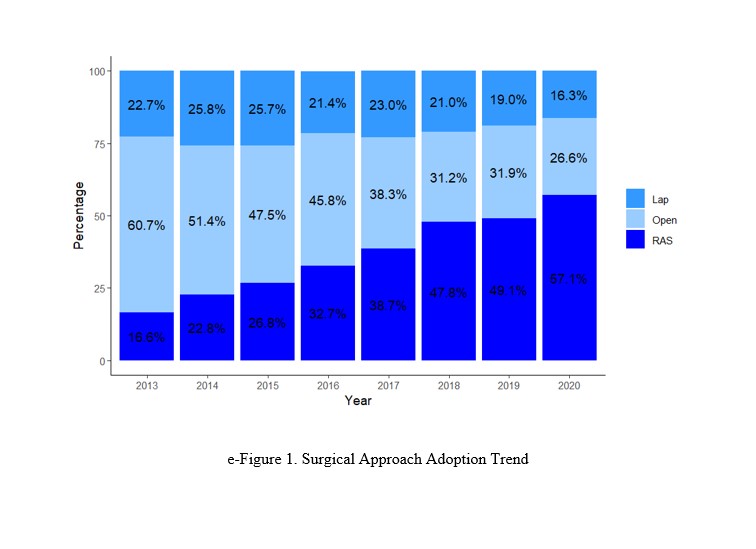

Supplement: Supplementary file 1 — Supplemental Figure 1. Surgical approach adoption trend Supplementary file1 (JPG 46 kb) [file 464_2024_10996_MOESM1_ESM.jpg]
