# Supplementary material for: Outcomes comparison of robotic-assisted versus laparoscopic and open surgery for patients undergoing rectal cancer resection with concurrent stoma creation
Source: Surg Endosc. 2024 Jun 28;38(8):4550–8. doi: 10.1007/s00464-024-10996-4 (PMC11289169; doi:10.1007/s00464-024-10996-4)
Supplement: Supplementary file 2 — Supplementary file2 (DOCX 17 kb) [file 464_2024_10996_MOESM2_ESM.docx]

eTable 1. Diagnosis and procedure codes used for patient identification and outcome definition

| Variables/Outcomes | Diagnosis and procedure Codes by Code Type | | |
| --- | --- | --- | --- |
|  | ICD-9 | ICD-10 | CPT/HCPCS |
| Rectum cancer | 154.1 | C20 |  |
| Surgical procedure^1^ |  |  |  |
| Rectal resection | 48.40, 48.41, 48.42, 48.43, 48.49, 48.63, 48.64, 48.65 | 0DTP0ZZ, 0DTP4ZZ | 44145, 44207, 45111, 45112, 45113, 45119, 45397 |
| Ileostomy formation | 46.01, 46.2, 46.21. 46.22, 46.23, 46.24 | 0D1B0Z4, 0D1B4Z4, 0D1B8Z4, 0D1B874,  0D1B8J4, 0D1B8K4 | 44310, 44187 |
| Resection with colostomy^2^/ colostomy formation | **48.50, 48.51, 48.52, 48.59, 48.62** 46.03, 46.10, 46.11, 46.13, 46.14 | 0D1N0Z4, 0D1N4Z4, 0D1N8Z4,  0D1M0Z4, 0D1M4Z4, 0D1M8Z4, 0D1L0Z4, 0D1L4Z4, 0D1L8Z4, 0D1K0Z4, 0D1K4Z4, 0D1K8Z4, 0D1E0Z4, 0D1E4Z4, 0D1E8Z4 | **44146, 44208, 45110, 45395** |
| Surgical approach |  |  |  |
| Robotic-assisted^3,4^ | **17.41, 17.42, 17.43, 17.44, 17.45, 17.49** | **8E0W0CZ, 8E0W3CZ, 8E0W4CZ,  8E0W7CZ, 8E0W8CZ, 8E0WXCZ** | **S2900** |
| Laparoscopic^4^ | 48.42, 48.51,  **54.21, 54.51** | 0DTP4ZZ | 44207, 45397, 44208, 45395 |

eTable 1. Continued

| Variables/Outcomes | Diagnosis and procedure Codes by Code Type | | |
| --- | --- | --- | --- |
|  | ICD-9 | ICD-10 | CPT/HCPCS |
| Anastomotic leak | 567.22, 569.81 | K63.2, K65.1 | - |
| Ileus | 560.1 | K56.0, K56.7 | - |
| Bleeding | 285.1, 459, 578.1, 578.9, 998.11, 998.12 | D62, K91.61, K91.62, K91.840, K91.841, K91.870, K91.871, K92.1, K92.2, R58 | - |
| Transfusion | 99.00, 99.01, 99.02, 99.03, 99.04, 99.05, 99.06, 99.07, 99.09 | 30233H0, 30233H1, 30233K1, 30233L1, 30233M1, 30233N0, 30233N1, 30233P1, 30233R1, 30243H0, 30243H1, 30243J1, 30243K1, 30243L1, 30243M1, 30243N0, 30243N1, 30243P1, 30243R1, 30253H0, 30253H1, 30253K1, 30253L1, 30253M1, 30253N0, 30253N1, 30253P1, 30253Q1, 30253R1, 30253T1, 30263H0, 30263H1, 30263K0, 30263K1, 30263L0, 30263L1, 30263M1, 30263N0, 30263N1, 30263P0, 30263P1, 30263R0, 30263R1 | 36430 |
| Surgical site infection | 567, 567.1, 567.21, 567.22, 567.29, 567.38, 567.89, 567.9, 569.5, 998.51, 998.59 | K63.0, K65.0, K65.1, K65.8, K65.9, K67, K68.11, K68.19, K68.9, T81.40XA, T81.40XD, T81.40XS, T81.41XA, T81.41XD, T81.41XS, T81.42XA, T81.42XD, T81.42XS, T81.43XA, T81.43XD, T81.43XS, T81.44XA, T81.44XD, T81.44XS, T81.49XA, T81.49XD, T81.49XS | - |
| Urinary retention | 788.2, 788.29 | R33.8, R33.9 | - |

^1^ Presence of a resection code along with ileostomy formation code or colostomy formation code was used to categorize patients into the resection with ileostomy cohort or the resection with colostomy cohort, respectively.

^2^ Patients with codes in **bold** were grouped into the resection with colostomy cohort, without requirement for additional colostomy code.

^3^ Codes in **bold** were used as modifier codes for robotic-assisted (RAS) or laparoscopic surgery (Lap) with a presence of surgical procedure code.

^4^ Hospital billing record were also examined using text-string search for identifying robotic procedures ('%ENDO%WRIST%', '%ENDO%WRST%', '%ENDWRST%', '%ENDOWRST%', '%ENDOWRIST%', '%ROBOT%', '%INTUITIVE%', '%VINCI%'), and hand-assist laparoscopic procedures ('%HANDPORT%', '%HAND%ASST%, '%GEL%PORT%, '%GELPORT%','%LAP%HAND%ASST%')

**Note**: Presence of RAS modifier code/text-string supersedes in defining surgical approach, followed by laparoscopic, and then open surgery.
