# Supplementary material for: Outcomes comparison of robotic-assisted versus laparoscopic and open surgery for patients undergoing rectal cancer resection with concurrent stoma creation
Source: Surg Endosc. 2024 Jun 28;38(8):4550–8. doi: 10.1007/s00464-024-10996-4 (PMC11289169; doi:10.1007/s00464-024-10996-4)
Supplement: Supplementary file 3 — Supplementary file3 (DOCX 18 kb) [file 464_2024_10996_MOESM3_ESM.docx]

eTable 2. Multivariable association between surgical approach and ileostomy versus colostomy formation among patients undergoing rectal cancer resection

| **Factors** | **O.R. (95% CI)** | **p-value** |
| --- | --- | --- |
| Surgical approach^1,2^ |  |  |
| RAS | 1.53 (1.39 to 1.69) | <.001 |
| Lap | 1.29 (1.16 to 1.43) | <.001 |
| Open | Reference |  |
| Age (years) |  |  |
| 18-44 | Reference |  |
| 45-54 | 0.87 (0.74 to 1.03) | 0.110 |
| 55-64 | 0.73 (0.62 to 0.85) | <.001 |
| 65+ | 0.56 (0.47 to 0.68) | <.001 |
| Sex |  |  |
| Female | Reference |  |
| Male | 0.97 (0.90 to 1.05) | 0.430 |
| Marital Status |  |  |
| Single | Reference |  |
| Married | 1.22 (1.12 to 1.32) | <.001 |
| Other | 1.04 (0.85 to 1.26) | 0.710 |
| Race/ Ethnicity |  |  |
| Black | Reference |  |
| White | 1.07 (0.92 to 1.23) | 0.390 |
| Hispanic | 1.09 (0.88 to 1.35) | 0.450 |
| Other | 1.30 (1.05 to 1.59) | 0.014 |
| Obese |  |  |
| Obese | 0.92 (0.83 to 1.03) | 0.140 |
| Not obese | Reference |  |
| Smoking |  |  |
| Smoking | 0.93 (0.86 to 1.00) | 0.061 |
| Not smoking |  |  |
| CCI score |  |  |
| 0 | Reference |  |
| 1 – 2 | 0.90 (0.82 to 0.98) | 0.022 |
| 3 – 4 | 0.80 (0.67 to 0.94) | 0.007 |
| 5+ | 0.65 (0.59 to 0.72) | <.001 |
| Payor Type |  |  |
| Commercial | Reference |  |
| Medicare | 0.74 (0.65 to 0.85) | <.001 |
| Medicaid | 0.69 (0.60 to 0.79) | <.001 |
| Other | 0.81 (0.68 to 0.97) | 0.019 |
| Surgeon Volume |  |  |
| Low volume | Reference |  |
| Medium volume | 1.12 (1.01 to 1.23) | 0.028 |
| High volume | 1.28 (1.14 to 1.43) | <.001 |
| Surgeon Specialty |  |  |
| Colorectal | Reference |  |
| General Surgery | 0.76 (0.68 to 0.86) | <.001 |
| Other | 0.85 (0.73 to 0.99) | 0.032 |
| Hospital Volume |  |  |
| Low volume | Reference |  |
| Medium volume | 1.04 (0.92 to 1.18) | 0.530 |
| High volume | 1.04 (0.89 to 1.23) | 0.620 |
| Teaching Hospital |  |  |
| Yes | 1.06 (0.92 to 1.24) | 0.410 |
| No | Reference |  |
| Hospital Bed Size |  |  |
| 000-300 | Reference |  |
| 300-499 | 1.03 (0.87 to 1.21) | 0.760 |
| 500+ | 1.03 (0.85 to 1.26) | 0.740 |
| Urban Hospital |  |  |
| Urban | 1.26 (1.01 to 1.58) | 0.039 |
| Rural | Reference |  |
| Hospital Region |  |  |
| Midwest | Reference |  |
| Northeast | 1.26 (1.01 to 1.57) | 0.039 |
| South | 1.00 (0.85 to 1.19) | 0.970 |
| West | 0.96 (0.78 to 1.17) | 0.660 |
| Surgery Year |  |  |
| 2013 | Reference |  |
| 2014 | 0.92 (0.80 to 1.07) | 0.290 |
| 2015 | 1.26 (1.08 to 1.46) | 0.003 |
| 2016 | 1.58 (1.34 to 1.85) | <.001 |
| 2017 | 1.47 (1.25 to 1.74) | <.001 |
| 2018 | 1.49 (1.26 to 1.76) | <.001 |
| 2019 | 1.50 (1.26 to 1.78) | <.001 |
| 2020 | 1.54 (1.28 to 1.84) | <.001 |

**Abbreviations**: RAS, robotic-assisted surgery; Lap, laparoscopic surgery; CCI, Charlson’s comorbidity index

^1^Using Lap as reference group the estimates would be: OR= 1.19, 95% CI= 1.06 to 1.33, p= 0.002 for RAS; and OR= 0.78, 95% CI= 0.70 to 0.86, p< .001 for Open.

^2^Using RAS as reference group the estimates would be: OR= 0.84, 95% CI= 0.75 to 0.94, p = 0.002 for Lap; and OR= 0.65, 95% CI= 0.59 to 0.72, p< .001 for Open.
