# Supplementary material for: Outcomes comparison of robotic-assisted versus laparoscopic and open surgery for patients undergoing rectal cancer resection with concurrent stoma creation
Source: Surg Endosc. 2024 Jun 28;38(8):4550–8. doi: 10.1007/s00464-024-10996-4 (PMC11289169; doi:10.1007/s00464-024-10996-4)
Supplement: Supplementary file 4 — Supplementary file4 (DOCX 30 kb) [file 464_2024_10996_MOESM4_ESM.docx]

eTable 3. Characteristics of rectum cancer resection patients with ileostomy formation: Before and after inverse-probability of treatment weighting comparison of robotic-assisted versus laparoscopic surgery

| Characteristics | Before IPTW |  | | |  | After IPTW | | |
| --- | --- | --- | --- | --- | --- | --- | --- | --- |
|  | Overall (n = 3,462) | Lap (n = 1,257) | RAS (n = 2,205) | *p* |  | Lap (n =1,835) | RAS (n =1,627) | *p* |
| Age, years |  |  |  | 0.110 |  |  |  | 0.910 |
| 18 – 44 | 322 (9.3) | 104 (8.3) | 218 (9.9) |  |  | 172 (9.4) | 157 (9.6) |  |
| 45 – 54 | 853 (24.6) | 311 (24.7) | 542 (24.6) |  |  | 439 (23.9) | 393 (24.2) |  |
| 55 – 64 | 1,120 (32.4) | 391 (31.1) | 729 (33.1) |  |  | 640 (34.9) | 535 (32.9) |  |
| 65+ | 1,167 (33.7) | 451 (35.9) | 716 (32.5) |  |  | 585 (31.9) | 541 (33.3) |  |
| Sex |  |  |  | 0.150 |  |  |  | 0.540 |
| Female | 1,257 (36.3) | 476 (37.9) | 781 (35.4) |  |  | 630 (34.4) | 586 (36.0) |  |
| Male | 2,205 (63.7) | 781 (62.1) | 1,424 (64.6) |  |  | 1,205 (65.6) | 1,040 (64.0) |  |
| Marital Status |  |  |  | 0.660 |  |  |  | 0.680 |
| Single | 1,176 (34.0) | 439 (34.9) | 737 (33.4) |  |  | 614 (33.4) | 545 (33.5) |  |
| Married | 2,040 (58.9) | 729 (58.0) | 1,311 (59.5) |  |  | 1,112 (60.6) | 967 (59.4) |  |
| Other | 246 (7.1) | 89 (7.1) | 157 (7.1) |  |  | 110 (6.0) | 115 (7.1) |  |
| Race/ethnicity |  |  |  | 0.330 |  |  |  | 0.860 |
| White | 2,730 (78.9) | 970 (77.2) | 1,760 (79.8) |  |  | 1,463 (79.7) | 1,275 (78.4) |  |
| Black | 266 (7.7) | 103 (8.2) | 163 (7.4) |  |  | 123 (6.7) | 122 (7.5) |  |
| Hispanic | 198 (5.7) | 79 (6.3) | 119 (5.4) |  |  | 113 (6.1) | 97 (6.0) |  |
| Other | 268 (7.7) | 105 (8.4) | 163 (7.4) |  |  | 137 (7.5) | 133 (8.2) |  |
| Obese/overweight | 561 (16.2) | 194 (15.4) | 367 (16.6) | 0.350 |  | 299 (16.3) | 268 (16.5) | 0.950 |
| Smoking history | 1,324 (38.2) | 476 (37.9) | 848 (38.5) | 0.730 |  | 743 (40.5) | 629 (38.7) | 0.530 |
| CCI score |  |  |  | **0.015** |  |  |  | 0.820 |
| 0 | 1,786 (51.6) | 639 (50.8) | 1,147 (52.0) |  |  | 930 (50.7) | 839 (51.6) |  |
| 1 – 2 | 971 (28.0) | 332 (26.4) | 639 (29.0) |  |  | 509 (27.7) | 459 (28.2) |  |
| 3 – 4 | 173 (5.0) | 61 (4.9) | 112 (5.1) |  |  | 85 (4.6) | 82 (5.1) |  |
| 5+ | 532 (15.4) | 225 (17.9) | 307 (13.9) |  |  | 312 (17.0) | 246 (15.1) |  |
| Payor type |  |  |  | 0.051 |  |  |  | 0.750 |
| Commercial | 1,801 (52.0) | 620 (49.3) | 1,181 (53.6) |  |  | 991 (54.0) | 854 (52.5) |  |
| Medicare | 1,169 (33.8) | 461 (36.7) | 708 (32.1) |  |  | 590 (32.2) | 539 (33.1) |  |
| Medicaid | 327 (9.4) | 117 (9.3) | 210 (9.5) |  |  | 151 (8.2) | 156 (9.6) |  |
| Other | 165 (4.8) | 59 (4.7) | 106 (4.8) |  |  | 103 (5.6) | 79 (4.8) |  |

eTable 3. Continued

| Variable | Before IPTW | | | |  | After IPTW | | |
| --- | --- | --- | --- | --- | --- | --- | --- | --- |
|  | Overall (n = 3,462) | Lap (n = 1,257) | RAS (n = 2,205) | *p* |  | Lap (n =1,835) | RAS (n =1,627) | *p* |
| Hospital location |  |  |  | **<.001** |  |  |  | 0.890 |
| Rural | 178 (5.1) | 86 (6.8) | 92 (4.2) |  |  | 84 (4.6) | 76 (4.7) |  |
| Urban | 3,284 (94.9) | 1,171 (93.2) | 2,113 (95.8) |  |  | 1,752 (95.4) | 1,551 (95.3) |  |
| Hospital region |  |  |  | **<.001** |  |  |  | 0.950 |
| Midwest | 873 (25.2) | 305 (24.3) | 568 (25.8) |  |  | 443 (24.1) | 412 (25.3) |  |
| Northeast | 591 (17.1) | 215 (17.1) | 376 (17.1) |  |  | 338 (18.4) | 287 (17.6) |  |
| South | 1,512 (43.7) | 515 (41.0) | 997 (45.2) |  |  | 817 (44.5) | 719 (44.2) |  |
| West | 486 (14.0) | 222 (17.7) | 264 (12.0) |  |  | 237 (12.9) | 210 (12.9) |  |
| Teaching hospital | 2,148 (62.0) | 747 (59.4) | 1,401 (63.5) | **0.017** |  | 1,156 (63.0) | 1,024 (63.0) | 0.990 |
| Hospital bed size |  |  |  | 0.110 |  |  |  | 0.680 |
| 000 – 299 | 673 (19.4) | 241 (19.2) | 432 (19.6) |  |  | 363 (19.8) | 309 (19.0) |  |
| 300 – 499 | 1,117 (32.3) | 381 (30.3) | 736 (33.4) |  |  | 566 (30.8) | 542 (33.3) |  |
| 500+ | 1,672 (48.3) | 635 (50.5) | 1,037 (47.0) |  |  | 907 (49.4) | 775 (47.7) |  |
| Hospital volume |  |  |  | **<.001** |  |  |  | **0.046** |
| Low | 718 (20.7) | 310 (24.7) | 408 (18.5) |  |  | 298 (16.2) | 310 (19.0) |  |
| Medium | 1,186 (34.3) | 402 (32.0) | 784 (35.6) |  |  | 570 (31.1) | 564 (34.7) |  |
| High | 1,558 (45.0) | 545 (43.4) | 1,013 (45.9) |  |  | 968 (52.7) | 753 (46.3) |  |
| Surgeon specialty |  |  |  | **<.001** |  |  |  | 0.880 |
| Colorectal | 1,947 (56.2) | 598 (47.6) | 1,349 (61.2) |  |  | 1,048 (57.1) | 944 (58.0) |  |
| General | 1,103 (31.9) | 469 (37.3) | 634 (28.8) |  |  | 569 (31.0) | 503 (30.9) |  |
| Other | 412 (11.9) | 190 (15.1) | 222 (10.1) |  |  | 218 (11.9) | 180 (11.1) |  |
| Surgeon volume |  |  |  | **0.001** |  |  |  | **0.011** |
| Low | 1,026 (29.6) | 330 (26.3) | 696 (31.6) |  |  | 690 (37.6) | 517 (31.8) |  |
| Medium | 1,176 (34.0) | 429 (34.1) | 747 (33.9) |  |  | 684 (37.2) | 576 (35.4) |  |
| High | 1,260 (36.4) | 498 (39.6) | 762 (34.6) |  |  | 462 (25.2) | 533 (32.8) |  |

eTable 3. Continued

| Variable | Before IPTW | | | |  | After IPTW | | |
| --- | --- | --- | --- | --- | --- | --- | --- | --- |
|  | Overall (n = 3,462) | Lap (n = 1,257) | RAS (n = 2,205) | *p* |  | Lap (n =1,835) | RAS (n =1,627) | *p* |
| Procedure year |  |  |  | **<.001** |  |  |  | 0.830 |
| 2013 | 261 (7.5) | 123 (9.8) | 138 (6.3) |  |  | 128 (7.0) | 115 (7.1) |  |
| 2014 | 348 (10.1) | 147 (11.7) | 201 (9.1) |  |  | 197 (10.7) | 164 (10.1) |  |
| 2015 | 435 (12.6) | 182 (14.5) | 253 (11.5) |  |  | 221 (12.0) | 197 (12.1) |  |
| 2016 | 440 (12.7) | 180 (14.3) | 260 (11.8) |  |  | 211 (11.5) | 194 (12.0) |  |
| 2017 | 490 (14.2) | 194 (15.4) | 296 (13.4) |  |  | 259 (14.1) | 228 (14.0) |  |
| 2018 | 535 (15.5) | 170 (13.5) | 365 (16.6) |  |  | 346 (18.9) | 257 (15.8) |  |
| 2019 | 498 (14.4) | 145 (11.5) | 353 (16.0) |  |  | 257 (14.0) | 242 (14.9) |  |
| 2020 | 455 (13.1) | 116 (9.2) | 339 (15.4) |  |  | 215 (11.7) | 229 (14.1) |  |

**Abbreviations**: RAS, robotic-assisted surgery; Lap, laparoscopic surgery; CCI, Charlson’s comorbidity index
