# Supplementary material for: Outcomes comparison of robotic-assisted versus laparoscopic and open surgery for patients undergoing rectal cancer resection with concurrent stoma creation
Source: Surg Endosc. 2024 Jun 28;38(8):4550–8. doi: 10.1007/s00464-024-10996-4 (PMC11289169; doi:10.1007/s00464-024-10996-4)
Supplement: Supplementary file 5 — Supplementary file5 (DOCX 30 kb) [file 464_2024_10996_MOESM5_ESM.docx]

eTable 4. Characteristics of rectum cancer resection patients with ileostomy formation: Before and after inverse-probability of treatment weighting comparison of robotic-assisted versus open surgery

| Characteristics | Before IPTW | | | |  | After IPTW | | |
| --- | --- | --- | --- | --- | --- | --- | --- | --- |
|  | Overall (n = 4,255) | Open (n = 2,050) | RAS (n = 2,205) | *p* |  | Open (n = 2,459) | RAS (n = 1,796) | *p* |
| Age, years |  |  |  | **<0.001** |  |  |  | 0.560 |
| 18 – 44 | 380 (8.9) | 162 (7.9) | 218 (9.9) |  |  | 180 (7.3) | 173 (9.6) |  |
| 45 – 54 | 1,005 (23.6) | 463 (22.6) | 542 (24.6) |  |  | 640 (26.0) | 426 (23.7) |  |
| 55 – 64 | 1,334 (31.4) | 605 (29.5) | 729 (33.1) |  |  | 794 (32.3) | 584 (32.5) |  |
| 65+ | 1,536 (36.1) | 820 (40.0) | 716 (32.5) |  |  | 845 (34.4) | 613 (34.1) |  |
| Sex |  |  |  | **0.033** |  |  |  | 0.960 |
| Female | 1,572 (36.9) | 791 (38.6) | 781 (35.4) |  |  | 896 (36.4) | 658 (36.6) |  |
| Male | 2,683 (63.1) | 1,259 (61.4) | 1,424 (64.6) |  |  | 1,563 (63.6) | 1,138 (63.4) |  |
| Marital Status |  |  |  | **<0.001** |  |  |  | 0.720 |
| Single | 1,512 (35.5) | 775 (37.8) | 737 (33.4) |  |  | 914 (37.2) | 620 (34.5) |  |
| Married | 2,402 (56.5) | 1,091 (53.2) | 1,311 (59.5) |  |  | 1,357 (55.2) | 1,039 (57.9) |  |
| Other | 341 (8.0) | 184 (9.0) | 157 (7.1) |  |  | 188 (7.6) | 136 (7.6) |  |
| Race/ethnicity |  |  |  | 0.17 |  |  |  | 0.620 |
| White | 3,359 (78.9) | 1,599 (78.0) | 1,760 (79.8) |  |  | 1,899 (77.2) | 1,422 (79.2) |  |
| Black | 306 (7.2) | 143 (7.0) | 163 (7.4) |  |  | 221 (9.0) | 123 (6.9) |  |
| Hispanic | 258 (6.1) | 139 (6.8) | 119 (5.4) |  |  | 126 (5.1) | 103 (5.7) |  |
| Other | 332 (7.8) | 169 (8.2) | 163 (7.4) |  |  | 214 (8.7) | 147 (8.2) |  |
| Obese/overweight | 664 (15.6) | 297 (14.5) | 367 (16.6) | 0.053 |  | 325 (13.2) | 264 (14.7) | 0.420 |
| Smoking history | 1,642 (38.6) | 794 (38.7) | 848 (38.5) | 0.85 |  | 1,082 (44.0) | 703 (39.1) | 0.170 |
| CCI score |  |  |  | **<0.001** |  |  |  | 0.740 |
| 0 | 2,150 (50.5) | 1,003 (48.9) | 1,147 (52.0) |  |  | 1,176 (47.8) | 920 (51.3) |  |
| 1 – 2 | 1,193 (28.0) | 554 (27.0) | 639 (29.0) |  |  | 747 (30.4) | 496 (27.6) |  |
| 3 – 4 | 227 (5.3) | 115 (5.6) | 112 (5.1) |  |  | 150 (6.1) | 96 (5.4) |  |
| 5+ | 685 (16.1) | 378 (18.4) | 307 (13.9) |  |  | 387 (15.7) | 283 (15.8) |  |
| Payor type |  |  |  | **<0.001** |  |  |  | 0.840 |
| Commercial | 2,107 (49.5) | 926 (45.2) | 1,181 (53.6) |  |  | 1,234 (50.2) | 893 (49.7) |  |
| Medicare | 1,518 (35.7) | 810 (39.5) | 708 (32.1) |  |  | 874 (35.6) | 613 (34.1) |  |
| Medicaid | 394 (9.3) | 184 (9.0) | 210 (9.5) |  |  | 235 (9.6) | 184 (10.2) |  |
| Other | 236 (5.5) | 130 (6.3) | 106 (4.8) |  |  | 116 (4.7) | 106 (5.9) |  |

eTable 4. Continued

| Variable | Before IPTW | | | |  | After IPTW | | |
| --- | --- | --- | --- | --- | --- | --- | --- | --- |
|  | Overall (n = 4,255) | Open (n = 2,050) | RAS (n = 2,205) | *p* |  | Open (n = 2,459) | RAS (n = 1,796) | *p* |
| Hospital location |  |  |  | **<0.001** |  |  |  | 0.900 |
| Rural | 244 (5.7) | 152 (7.4) | 92 (4.2) |  |  | 105 (4.3) | 75 (4.2) |  |
| Urban | 4,011 (94.3) | 1,898 (92.6) | 2,113 (95.8) |  |  | 2,354 (95.7) | 1,720 (95.8) |  |
| Hospital region |  |  |  | **<0.001** |  |  |  | 0.370 |
| Midwest | 1,044 (24.5) | 476 (23.2) | 568 (25.8) |  |  | 668 (27.2) | 434 (24.2) |  |
| Northeast | 624 (14.7) | 248 (12.1) | 376 (17.1) |  |  | 340 (13.8) | 315 (17.5) |  |
| South | 1,991 (46.8) | 994 (48.5) | 997 (45.2) |  |  | 1,167 (47.4) | 830 (46.2) |  |
| West | 596 (14.0) | 332 (16.2) | 264 (12.0) |  |  | 285 (11.6) | 217 (12.1) |  |
| Teaching hospital | 2,606 (61.2) | 1,205 (58.8) | 1,401 (63.5) | **0.001** |  | 1,515 (61.6) | 1,174 (65.4) | 0.270 |
| Hospital bed size |  |  |  | 0.54 |  |  |  | 0.410 |
| 000 – 299 | 835 (19.6) | 403 (19.7) | 432 (19.6) |  |  | 374 (15.2) | 320 (17.8) |  |
| 300 – 499 | 1,389 (32.6) | 653 (31.9) | 736 (33.4) |  |  | 895 (36.4) | 595 (33.1) |  |
| 500+ | 2,031 (47.7) | 994 (48.5) | 1,037 (47.0) |  |  | 1,191 (48.4) | 881 (49.0) |  |
| Hospital volume |  |  |  | **<0.001** |  |  |  | 0.590 |
| Low | 1,039 (24.4) | 631 (30.8) | 408 (18.5) |  |  | 478 (19.4) | 395 (22.0) |  |
| Medium | 1,451 (34.1) | 667 (32.5) | 784 (35.6) |  |  | 875 (35.6) | 602 (33.6) |  |
| High | 1,765 (41.5) | 752 (36.7) | 1,013 (45.9) |  |  | 1,106 (45.0) | 799 (44.5) |  |
| Surgeon specialty |  |  |  | **<0.001** |  |  |  | 0.320 |
| Colorectal | 2,392 (56.2) | 1,043 (50.9) | 1,349 (61.2) |  |  | 1,363 (55.4) | 1,000 (55.7) |  |
| General | 1,374 (32.3) | 740 (36.1) | 634 (28.8) |  |  | 830 (33.7) | 550 (30.6) |  |
| Other | 489 (11.5) | 267 (13.0) | 222 (10.1) |  |  | 266 (10.8) | 245 (13.7) |  |
| Surgeon volume |  |  |  | **<0.001** |  |  |  | 0.640 |
| Low | 1,208 (28.4) | 512 (25.0) | 696 (31.6) |  |  | 845 (34.4) | 622 (34.7) |  |
| Medium | 1,392 (32.7) | 645 (31.5) | 747 (33.9) |  |  | 849 (34.5) | 573 (31.9) |  |
| High | 1,655 (38.9) | 893 (43.6) | 762 (34.6) |  |  | 766 (31.1) | 601 (33.4) |  |

eTable 4. Continued

| Variable | Before IPTW | | | |  | After IPTW | | |
| --- | --- | --- | --- | --- | --- | --- | --- | --- |
|  | Overall (n = 4,255) | Open (n = 2,050) | RAS (n = 2,205) | *p* |  | Open (n = 2,459) | RAS (n = 1,796) | *p* |
| Procedure year |  |  |  | **<0.001** |  |  |  | 0.850 |
| 2013 | 551 (12.9) | 413 (20.1) | 138 (6.3) |  |  | 288 (11.7) | 219 (12.2) |  |
| 2014 | 605 (14.2) | 404 (19.7) | 201 (9.1) |  |  | 308 (12.5) | 218 (12.1) |  |
| 2015 | 644 (15.1) | 391 (19.1) | 253 (11.5) |  |  | 325 (13.2) | 235 (13.1) |  |
| 2016 | 491 (11.5) | 231 (11.3) | 260 (11.8) |  |  | 255 (10.4) | 203 (11.3) |  |
| 2017 | 490 (11.5) | 194 (9.5) | 296 (13.4) |  |  | 367 (14.9) | 216 (12.0) |  |
| 2018 | 515 (12.1) | 150 (7.3) | 365 (16.6) |  |  | 307 (12.5) | 253 (14.1) |  |
| 2019 | 521 (12.2) | 168 (8.2) | 353 (16.0) |  |  | 283 (11.5) | 239 (13.3) |  |
| 2020 | 438 (10.3) | 99 (4.8) | 339 (15.4) |  |  | 326 (13.3) | 213 (11.9) |  |

**Abbreviations**: RAS, robotic-assisted surgery; Lap, laparoscopic surgery; CCI, Charlson’s comorbidity index
