# Supplementary material for: Outcomes comparison of robotic-assisted versus laparoscopic and open surgery for patients undergoing rectal cancer resection with concurrent stoma creation
Source: Surg Endosc. 2024 Jun 28;38(8):4550–8. doi: 10.1007/s00464-024-10996-4 (PMC11289169; doi:10.1007/s00464-024-10996-4)
Supplement: Supplementary file 6 — Supplementary file6 (DOCX 30 kb) [file 464_2024_10996_MOESM6_ESM.docx]

eTable 5. Characteristics of rectum cancer resection patients with colostomy formation: Before and after inverse-probability of treatment weighting comparison of robotic-assisted versus laparoscopic surgery

| Characteristics | Before IPTW |  | | |  | After IPTW | | |
| --- | --- | --- | --- | --- | --- | --- | --- | --- |
|  | Overall (n = 3,726) | Lap (n = 1,615) | RAS (n = 2,111) | *p* |  | Lap (n = 2,017) | RAS (n = 1,709) | *p* |
| Age, years |  |  |  | 0.870 |  |  |  | 0.480 |
| 18 – 44 | 223 (6.0) | 98 (6.1) | 125 (5.9) |  |  | 96 (4.8) | 100 (5.9) |  |
| 45 – 54 | 668 (17.9) | 281 (17.4) | 387 (18.3) |  |  | 333 (16.5) | 311 (18.2) |  |
| 55 – 64 | 1,009 (27.1) | 435 (26.9) | 574 (27.2) |  |  | 510 (25.3) | 449 (26.3) |  |
| 65+ | 1,826 (49.0) | 801 (49.6) | 1,025 (48.6) |  |  | 1,078 (53.4) | 849 (49.7) |  |
| Sex |  |  |  | 0.590 |  |  |  | 0.710 |
| Female | 1,357 (36.4) | 596 (36.9) | 761 (36.0) |  |  | 757 (37.5) | 620 (36.3) |  |
| Male | 2,369 (63.6) | 1,019 (63.1) | 1,350 (64.0) |  |  | 1,260 (62.5) | 1,088 (63.7) |  |
| Marital Status |  |  |  | **<.001** |  |  |  | 0.520 |
| Single | 1,516 (40.7) | 625 (38.7) | 891 (42.2) |  |  | 772 (38.3) | 703 (41.1) |  |
| Married | 1,972 (52.9) | 843 (52.2) | 1,129 (53.5) |  |  | 1,133 (56.1) | 913 (53.4) |  |
| Other | 238 (6.4) | 147 (9.1) | 91 (4.3) |  |  | 113 (5.6) | 93 (5.5) |  |
| Race/ethnicity |  |  |  | **0.036** |  |  |  | 0.520 |
| White | 3,010 (80.8) | 1,294 (80.1) | 1,716 (81.3) |  |  | 1,673 (82.9) | 1,375 (80.5) |  |
| Black | 279 (7.5) | 109 (6.7) | 170 (8.1) |  |  | 133 (6.6) | 131 (7.7) |  |
| Hispanic | 199 (5.3) | 103 (6.4) | 96 (4.5) |  |  | 82 (4.1) | 88 (5.1) |  |
| Other | 238 (6.4) | 109 (6.7) | 129 (6.1) |  |  | 130 (6.4) | 114 (6.7) |  |
| Obese/overweight | 611 (16.4) | 263 (16.3) | 348 (16.5) | 0.870 |  | 264 (13.1) | 266 (15.6) | 0.130 |
| Smoking history | 1,543 (41.4) | 643 (39.8) | 900 (42.6) | 0.083 |  | 825 (40.9) | 706 (41.3) | 0.900 |
| CCI score |  |  |  | 0.520 |  |  |  | 0.560 |
| 0 | 1,640 (44.0) | 729 (45.1) | 911 (43.2) |  |  | 952 (47.2) | 750 (43.9) |  |
| 1 – 2 | 1,069 (28.7) | 444 (27.5) | 625 (29.6) |  |  | 517 (25.6) | 491 (28.7) |  |
| 3 – 4 | 258 (6.9) | 111 (6.9) | 147 (7.0) |  |  | 155 (7.7) | 115 (6.8) |  |
| 5+ | 759 (20.4) | 331 (20.5) | 428 (20.3) |  |  | 393 (19.5) | 352 (20.6) |  |
| Payor type |  |  |  | 0.660 |  |  |  | 0.460 |
| Commercial | 1,370 (36.8) | 579 (35.9) | 791 (37.5) |  |  | 751 (37.3) | 631 (36.9) |  |
| Medicare | 1,809 (48.6) | 794 (49.2) | 1,015 (48.1) |  |  | 1,029 (51.0) | 837 (49.0) |  |
| Medicaid | 337 (9.0) | 145 (9.0) | 192 (9.1) |  |  | 131 (6.5) | 148 (8.6) |  |
| Other | 210 (5.6) | 97 (6.0) | 113 (5.4) |  |  | 106 (5.3) | 94 (5.5) |  |

eTable 5. Continued

| Variable | Before IPTW | | | |  | After IPTW | | |
| --- | --- | --- | --- | --- | --- | --- | --- | --- |
|  | Overall (n = 3,726) | Lap (n = 1,615) | RAS (n = 2,111) | *p* |  | Lap (n = 2,017) | RAS (n = 1,709) | *p* |
| Hospital location |  |  |  | **0.002** |  |  |  | 0.720 |
| Rural | 259 (7.0) | 136 (8.4) | 123 (5.8) |  |  | 121 (6.0) | 109 (6.4) |  |
| Urban | 3,467 (93.0) | 1,479 (91.6) | 1,988 (94.2) |  |  | 1,896 (94.0) | 1,600 (93.6) |  |
| Hospital region |  |  |  | **<.001** |  |  |  | 0.500 |
| Midwest | 871 (23.4) | 324 (20.1) | 547 (25.9) |  |  | 458 (22.7) | 405 (23.7) |  |
| Northeast | 541 (14.5) | 232 (14.4) | 309 (14.6) |  |  | 259 (12.8) | 264 (15.4) |  |
| South | 1,673 (44.9) | 689 (42.7) | 984 (46.6) |  |  | 960 (47.6) | 785 (45.9) |  |
| West | 641 (17.2) | 370 (22.9) | 271 (12.8) |  |  | 340 (16.8) | 255 (14.9) |  |
| Teaching hospital | 2,127 (57.1) | 839 (52.0) | 1,288 (61.0) | **<.001** |  | 1,196 (59.3) | 1,041 (60.9) | 0.580 |
| Hospital bed size |  |  |  | **<.001** |  |  |  | 0.350 |
| 000 – 299 | 829 (22.2) | 401 (24.8) | 428 (20.3) |  |  | 357 (17.7) | 358 (20.9) |  |
| 300 – 499 | 1,169 (31.4) | 516 (32.0) | 653 (30.9) |  |  | 654 (32.4) | 534 (31.2) |  |
| 500+ | 1,728 (46.4) | 698 (43.2) | 1,030 (48.8) |  |  | 1,007 (49.9) | 817 (47.8) |  |
| Hospital volume |  |  |  | **<.001** |  |  |  | 0.510 |
| Low | 976 (26.2) | 550 (34.1) | 426 (20.2) |  |  | 467 (23.2) | 418 (24.4) |  |
| Medium | 1,293 (34.7) | 539 (33.4) | 754 (35.7) |  |  | 650 (32.2) | 586 (34.3) |  |
| High | 1,457 (39.1) | 526 (32.6) | 931 (44.1) |  |  | 900 (44.6) | 705 (41.3) |  |
| Surgeon specialty |  |  |  | **<.001** |  |  |  | 0.310 |
| Colorectal | 1,940 (52.1) | 773 (47.9) | 1,167 (55.3) |  |  | 1,117 (55.4) | 905 (53.0) |  |
| General | 1,262 (33.9) | 627 (38.8) | 635 (30.1) |  |  | 661 (32.8) | 550 (32.2) |  |
| Other | 524 (14.1) | 215 (13.3) | 309 (14.6) |  |  | 240 (11.9) | 253 (14.8) |  |
| Surgeon volume |  |  |  | 0.580 |  |  |  | 0.052 |
| Low | 1,321 (35.5) | 586 (36.3) | 735 (34.8) |  |  | 791 (39.2) | 656 (38.4) |  |
| Medium | 1,250 (33.5) | 529 (32.8) | 721 (34.2) |  |  | 792 (39.3) | 581 (34.0) |  |
| High | 1,155 (31.0) | 500 (31.0) | 655 (31.0) |  |  | 435 (21.5) | 472 (27.6) |  |

eTable 5. Continued

| Variable | Before IPTW | | | |  | After IPTW | | |
| --- | --- | --- | --- | --- | --- | --- | --- | --- |
|  | Overall (n = 3,726) | Lap (n = 1,615) | RAS (n = 2,111) | *p* |  | Lap (n = 2,017) | RAS (n = 1,709) | *p* |
| Procedure year |  |  |  | **<.001** |  |  |  | 0.520 |
| 2013 | 513 (13.8) | 324 (20.1) | 189 (9.0) |  |  | 258 (12.8) | 233 (13.7) |  |
| 2014 | 702 (18.8) | 411 (25.4) | 291 (13.8) |  |  | 358 (17.7) | 304 (17.8) |  |
| 2015 | 603 (16.2) | 326 (20.2) | 277 (13.1) |  |  | 305 (15.1) | 256 (15.0) |  |
| 2016 | 331 (8.9) | 125 (7.7) | 206 (9.8) |  |  | 143 (7.1) | 146 (8.5) |  |
| 2017 | 398 (10.7) | 137 (8.5) | 261 (12.4) |  |  | 218 (10.8) | 187 (11.0) |  |
| 2018 | 420 (11.3) | 121 (7.5) | 299 (14.2) |  |  | 208 (10.3) | 202 (11.8) |  |
| 2019 | 409 (11.0) | 108 (6.7) | 301 (14.3) |  |  | 223 (11.1) | 202 (11.8) |  |
| 2020 | 350 (9.4) | 63 (3.9) | 287 (13.6) |  |  | 305 (15.1) | 178 (10.4) |  |

**Abbreviations**: RAS, robotic-assisted surgery; Lap, laparoscopic surgery; CCI, Charlson’s comorbidity index
