# Supplementary material for: Outcomes comparison of robotic-assisted versus laparoscopic and open surgery for patients undergoing rectal cancer resection with concurrent stoma creation
Source: Surg Endosc. 2024 Jun 28;38(8):4550–8. doi: 10.1007/s00464-024-10996-4 (PMC11289169; doi:10.1007/s00464-024-10996-4)
Supplement: Supplementary file 7 — Supplementary file7 (DOCX 30 kb) [file 464_2024_10996_MOESM7_ESM.docx]

eTable 6. Characteristics of rectum cancer resection patients with colostomy formation: Before and after inverse-probability of treatment weighting comparison of robotic-assisted versus open surgery

| Characteristics | Before IPTW | | | |  | After IPTW | | |
| --- | --- | --- | --- | --- | --- | --- | --- | --- |
|  | Overall (n = 5,660) | Open (n = 3,549) | RAS (n = 2,111) | *p* |  | Open (n = 3,208) | RAS (n = 2,452) | *p* |
| Age, years |  |  |  | 0.080 |  |  |  | 0.590 |
| 18 – 44 | 302 (5.3) | 177 (5.0) | 125 (5.9) |  |  | 149 (4.6) | 144 (5.9) |  |
| 45 – 54 | 969 (17.1) | 582 (16.4) | 387 (18.3) |  |  | 563 (17.6) | 405 (16.5) |  |
| 55 – 64 | 1,599 (28.3) | 1,025 (28.9) | 574 (27.2) |  |  | 874 (27.3) | 679 (27.7) |  |
| 65+ | 2,790 (49.3) | 1,765 (49.7) | 1,025 (48.6) |  |  | 1,622 (50.6) | 1,224 (49.9) |  |
| Sex |  |  |  | 0.740 |  |  |  | 0.850 |
| Female | 2,056 (36.3) | 1,295 (36.5) | 761 (36.0) |  |  | 1,218 (38.0) | 920 (37.5) |  |
| Male | 3,604 (63.7) | 2,254 (63.5) | 1,350 (64.0) |  |  | 1,990 (62.0) | 1,532 (62.5) |  |
| Marital Status |  |  |  | **<.001** |  |  |  | 0.780 |
| Single | 2,393 (42.3) | 1,502 (42.3) | 891 (42.2) |  |  | 1,369 (42.7) | 1,046 (42.7) |  |
| Married | 2,867 (50.7) | 1,738 (49.0) | 1,129 (53.5) |  |  | 1,623 (50.6) | 1,260 (51.4) |  |
| Other | 400 (7.1) | 309 (8.7) | 91 (4.3) |  |  | 216 (6.7) | 146 (5.9) |  |
| Race/ethnicity |  |  |  | **0.004** |  |  |  | 0.750 |
| White | 4,463 (78.9) | 2,747 (77.4) | 1,716 (81.3) |  |  | 2,555 (79.6) | 1,944 (79.3) |  |
| Black | 484 (8.6) | 314 (8.8) | 170 (8.1) |  |  | 273 (8.5) | 203 (8.3) |  |
| Hispanic | 307 (5.4) | 211 (5.9) | 96 (4.5) |  |  | 163 (5.1) | 114 (4.6) |  |
| Other | 406 (7.2) | 277 (7.8) | 129 (6.1) |  |  | 217 (6.8) | 191 (7.8) |  |
| Obese/overweight | 860 (15.2) | 512 (14.4) | 348 (16.5) | **0.037** |  | 447 (13.9) | 355 (14.5) | 0.670 |
| Smoking history | 2,368 (41.8) | 1,468 (41.4) | 900 (42.6) | 0.350 |  | 1,336 (41.7) | 1,022 (41.7) | >0.99 |
| CCI score |  |  |  | **0.004** |  |  |  | 0.790 |
| 0 | 2,321 (41.0) | 1,410 (39.7) | 911 (43.2) |  |  | 1,308 (40.8) | 971 (39.6) |  |
| 1 – 2 | 1,647 (29.1) | 1,022 (28.8) | 625 (29.6) |  |  | 981 (30.6) | 733 (29.9) |  |
| 3 – 4 | 402 (7.1) | 255 (7.2) | 147 (7.0) |  |  | 201 (6.3) | 159 (6.5) |  |
| 5+ | 1,290 (22.8) | 862 (24.3) | 428 (20.3) |  |  | 717 (22.4) | 590 (24.0) |  |
| Payor type |  |  |  | **<.001** |  |  |  | 0.770 |
| Commercial | 1,947 (34.4) | 1,156 (32.6) | 791 (37.5) |  |  | 1,081 (33.7) | 868 (35.4) |  |
| Medicare | 2,808 (49.6) | 1,793 (50.5) | 1,015 (48.1) |  |  | 1,637 (51.0) | 1,233 (50.3) |  |
| Medicaid | 610 (10.8) | 418 (11.8) | 192 (9.1) |  |  | 321 (10.0) | 223 (9.1) |  |
| Other | 295 (5.2) | 182 (5.1) | 113 (5.4) |  |  | 168 (5.2) | 128 (5.2) |  |

eTable 6. Continued

| Variable | Before IPTW | | | |  | After IPTW | | |
| --- | --- | --- | --- | --- | --- | --- | --- | --- |
|  | Overall (n = 5,660) | Open (n = 3,549) | RAS (n = 2,111) | *p* |  | Open (n = 3,208) | RAS (n = 2,452) | *p* |
| Hospital location |  |  |  | **<.001** |  |  |  | 0.190 |
| Rural | 515 (9.1) | 392 (11.0) | 123 (5.8) |  |  | 263 (8.2) | 168 (6.9) |  |
| Urban | 5,145 (90.9) | 3,157 (89.0) | 1,988 (94.2) |  |  | 2,945 (91.8) | 2,284 (93.1) |  |
| Hospital region |  |  |  | **<.001** |  |  |  | 0.940 |
| Midwest | 1,339 (23.7) | 792 (22.3) | 547 (25.9) |  |  | 760 (23.7) | 573 (23.4) |  |
| Northeast | 677 (12.0) | 368 (10.4) | 309 (14.6) |  |  | 443 (13.8) | 361 (14.7) |  |
| South | 2,708 (47.8) | 1,724 (48.6) | 984 (46.6) |  |  | 1,488 (46.4) | 1,134 (46.2) |  |
| West | 936 (16.5) | 665 (18.7) | 271 (12.8) |  |  | 518 (16.1) | 384 (15.7) |  |
| Teaching hospital | 3,238 (57.2) | 1,950 (54.9) | 1,288 (61.0) | **<.001** |  | 1,854 (57.8) | 1,487 (60.6) | 0.210 |
| Hospital bed size |  |  |  | **<.001** |  |  |  | 0.058 |
| 000 – 299 | 1,292 (22.8) | 864 (24.3) | 428 (20.3) |  |  | 753 (23.5) | 508 (20.7) |  |
| 300 – 499 | 1,844 (32.6) | 1,191 (33.6) | 653 (30.9) |  |  | 1,075 (33.5) | 752 (30.7) |  |
| 500+ | 2,524 (44.6) | 1,494 (42.1) | 1,030 (48.8) |  |  | 1,379 (43.0) | 1,192 (48.6) |  |
| Hospital volume |  |  |  | **<.001** |  |  |  | 0.390 |
| Low | 1,791 (31.6) | 1,365 (38.5) | 426 (20.2) |  |  | 930 (29.0) | 659 (26.9) |  |
| Medium | 1,878 (33.2) | 1,124 (31.7) | 754 (35.7) |  |  | 1,096 (34.2) | 820 (33.4) |  |
| High | 1,991 (35.2) | 1,060 (29.9) | 931 (44.1) |  |  | 1,182 (36.8) | 973 (39.7) |  |
| Surgeon specialty |  |  |  | **<.001** |  |  |  | 0.540 |
| Colorectal | 2,518 (44.5) | 1,351 (38.1) | 1,167 (55.3) |  |  | 1,458 (45.5) | 1,125 (45.9) |  |
| General | 2,348 (41.5) | 1,713 (48.3) | 635 (30.1) |  |  | 1,304 (40.6) | 950 (38.8) |  |
| Other | 794 (14.0) | 485 (13.7) | 309 (14.6) |  |  | 445 (13.9) | 377 (15.4) |  |
| Surgeon volume |  |  |  | **0.012** |  |  |  | 0.360 |
| Low | 1,872 (33.1) | 1,137 (32.0) | 735 (34.8) |  |  | 1,137 (35.5) | 943 (38.5) |  |
| Medium | 1,901 (33.6) | 1,180 (33.2) | 721 (34.2) |  |  | 1,086 (33.8) | 804 (32.8) |  |
| High | 1,887 (33.3) | 1,232 (34.7) | 655 (31.0) |  |  | 985 (30.7) | 705 (28.8) |  |

eTable 6. Continued

| Variable | Before IPTW | | | |  | After IPTW | | |
| --- | --- | --- | --- | --- | --- | --- | --- | --- |
|  | Overall (n = 5,660) | Open (n = 3,549) | RAS (n = 2,111) | *p* |  | Open (n = 3,208) | RAS (n = 2,452) | *p* |
| Procedure year |  |  |  | **<.001** |  |  |  | 0.810 |
| 2013 | 971 (17.2) | 782 (22.0) | 189 (9.0) |  |  | 525 (16.4) | 426 (17.4) |  |
| 2014 | 998 (17.6) | 707 (19.9) | 291 (13.8) |  |  | 522 (16.3) | 373 (15.2) |  |
| 2015 | 827 (14.6) | 550 (15.5) | 277 (13.1) |  |  | 441 (13.7) | 340 (13.9) |  |
| 2016 | 627 (11.1) | 421 (11.9) | 206 (9.8) |  |  | 340 (10.6) | 259 (10.6) |  |
| 2017 | 618 (10.9) | 357 (10.1) | 261 (12.4) |  |  | 335 (10.4) | 276 (11.2) |  |
| 2018 | 582 (10.3) | 283 (8.0) | 299 (14.2) |  |  | 372 (11.6) | 276 (11.2) |  |
| 2019 | 557 (9.8) | 256 (7.2) | 301 (14.3) |  |  | 405 (12.6) | 265 (10.8) |  |
| 2020 | 480 (8.5) | 193 (5.4) | 287 (13.6) |  |  | 269 (8.4) | 237 (9.7) |  |

**Abbreviations**: RAS, robotic-assisted surgery; Lap, laparoscopic surgery; CCI, Charlson’s comorbidity index
